# Silicon-on-Insulator Optical Waveguide Pressure Sensor Based on Mach-Zehnder Interferometer

**DOI:** 10.3390/mi13081321

**Published:** 2022-08-15

**Authors:** Chen Li, Chi Zhang, Lijun Yang, Fangtong Guo

**Affiliations:** 1 School of Mechanical and Electrical Engineering, Shaanxi University of Science and Technology, Xi’an 710021, China; 2 School of Mechanical Engineering, Xi’an Jiaotong University, Xi’an 710049, China; 3 State Key Laboratory of Mechanical Manufacturing System Engineering, Xi’an Jiaotong University, Xi’an 710054, China; 4 Peking University Founder Technology College, Beijing 065000, China

**Keywords:** silicon on insulator, pressure sensor, Mach–Zehnder interferometer, directional coupler, ridge waveguide

## Abstract

At present, there are few methods to measure optical pressure using MEMS. However, due to its high precision and fast response, a ridge waveguide pressure sensor based on a Mach–Zehnder interferometer is designed in this paper. Through the design and optimization of each component of the structure, the sensitivity of the pressure sensor was 2.2 × 10^−3^ W/kPa and the linearity was 5.9 × 10^−3^. The sensor had a good performance and small volume, which can be used in the field of light pressure measurement and other fields that required the measurement small pressures, such as the biomedicine field.

## 1. Introduction

Light pressure is the pressure of light on the surface of an object, which was first proposed by Maxwell [1,2]. Although photons have no rest mass, they have momentum, and laser irradiation on the surface of an object will generate pressure. The existing methods of measuring light pressure include the torsion balance method [3], flat capacitance method [4,5,6,7], thin film method [8], and liquid method [9,10,11], etc. Compared with the methods mentioned above, the MEMS (micro-electro-mechanical system) method for measuring optical pressure has the advantage of fast response, real-time measurement, and high accuracy, and has become a research hotspot [12].

MEMSs have developed rapidly and have been widely used in many fields, due to their low-cost stable performance and ease of manufacture [13]. A micro-optical-electro-mechanical system (MOEMS) is a highly dynamic new technology system developed in MEMS, in recent years. It is a new type of micro-optical structure system produced by the combination of micro-optics, microelectronics, and micro-machines. Due to its small size, small power consumption, good ability to resist electromagnetic interference, capability, large operating bandwidth, and high sensitivity, it has gained more and more attention [14]. Currently, traditional pressure sensors have difficulties in meeting the requirements for pressure sensors, due to their large volume and unstable performance. For example, in the biomedical field, there are many situations that require small pressure measurement, such as deep venous thrombosis, leg ulcers, varicose veins, and hypertrophic scars. The pressure range is 0–6.5 kPa [15]; there are medical sensors for direct measurement of pressure flow in blood vessels [16], as well as, medical measurement of intraocular pressure and intracranial pressure, with pressure ranging from 0 to 3 kPa [17]. Recently, the photo-elastic effect and MZI principle have been used to fabricate pressure sensors [18]. For example, Abeysinghe proposed a novel MEMS pressure sensor fabricated on optical fiber in 2001 [19]; Rochus proposed a MZI micro-opto-mechanical pressure sensor (MOMPS) for the SiN Photonics Platform [20]. Considering the wider application of SOI in MEMS sensors, we propose a SOI ridge waveguide pressure sensor based on MZI.

## 2. Principle of Pressure Sensors

**Figure 1:**
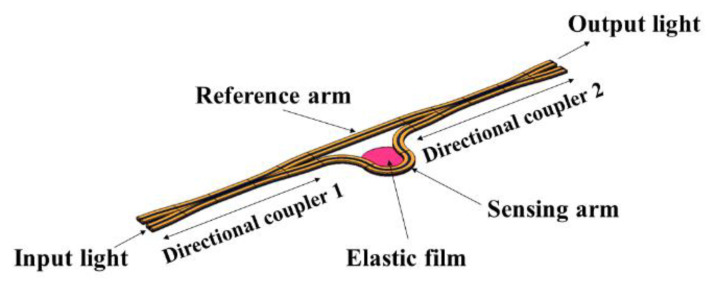
Pressure sensor structure diagram.

A pressure sensor is composed of an optical waveguide structure and an elastic diaphragm. Silicon-on-insulator (SOI) materials are used in the optical waveguide structure, which is composed of two directional couplers and two waveguide arms. SOI is composed of a substrate made up of silicon (Si), on top of which is a thin Si film on a silica (SiO_2_) buried layer. The cladding material is SiO_2_ and the core material is Si. The elastic film material is aluminum. A structure diagram is shown in Figure 1.

The working principle of a pressure sensor is as follows. First, the input laser with a wavelength of 1550 nm is evenly distributed to the sensing arm and the reference arm, after passing through the directional coupler 1, then the synthesized light is output from the port through the directional coupler 2. When the light pressure acts on the elastic film, the radiation pressure will cause the deformation of the elastic film, resulting in an optical path change in the sensing arm. At the same time, based on the photo-elastic effect, the external light pressure changes the optical phase of the sensing arm, thereby changing the output laser intensity. According to the relationship between the external light pressure and the output laser intensity, the specific size of the external load is measured.

## 3. Optimization of an MZI Ridge Optical Waveguide

### 3.1. Design of a Ridge Optical Waveguide

The transmission efficiency of an optical waveguide is an extremely important factor affecting the performance of pressure sensors. The ridge-shaped optical waveguide is made of SOI material, because the thickness of the top Si is relatively large, which can be used to make large cross-sectional sized optical waveguides and thus reduce the coupling loss with optical fibers [21]. A ridge structure can also effectively reduce the coupling loss, and is one of the most commonly used waveguide structures in integrated optics. 

**Figure 2:**
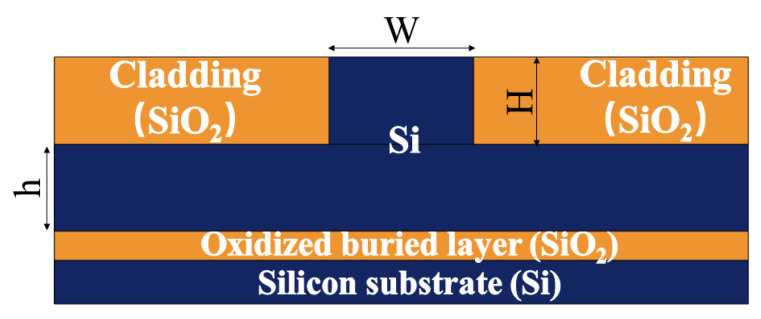
Schematic diagram of an SOI ridge waveguide.

Figure 2 shows the selected SOI ridge waveguide structure, whose single mode condition is expressed as follows [22,23,24]:

(1) $$\left\{\begin{array}{l} r>0.5\\ t<\frac{r}{{\left(1-r^{2}\right)}^{\frac{1}{2}}}+c_{1} \end{array}\right.$$

where *c*_1_ is a coefficient constant and *c*_1_ = 0.3, *t* = *W*_eff_/*H*_eff_, *r* = *h*_eff_/*H*_eff_. Where *W*_eff_ is the equivalent ridge width, *H*_eff_ is the equivalent ridge height, *h*_eff_ is the external equivalent ridge height. These can be obtained from the following equation:

(2) $$\left\{\begin{array}{l} W_{eff}\leq W+\frac{2\gamma_{1}}{k_{0}\sqrt{n_{2}^{2}-n_{1}^{2}}}\\ h_{eff}=h+\frac{q}{k_{0}}\\ H_{eff}=H+\frac{q}{k_{0}}\\ q=\frac{\gamma_{1}}{\sqrt{n_{2}^{2}-n_{1}^{2}}}+\frac{\gamma_{3}}{\sqrt{n_{2}^{2}-n_{3}^{2}}} \end{array}\right.$$

where *n*_1_, *n*_2_, and *n*_3_ are the waveguide outer cladding, core, and refractive index of the insulating layer, *n*_1_ = *n*_3_ = 1.445, *n*_2_ = 3.445, *k*_0_ = 2π/λ.

(3) $$\gamma_{1,3}=\left\{\begin{array}{l} 1,\;TE\;\\ \frac{n_{1,3}}{n_{2}},\;TM \end{array}\right.$$

For an SOI waveguide, because n_2_ is much larger than n_1_ and n_3_, *w*_eff_ ≈ *W*, *h*_eff_ ≈ *h*, and *H*_eff_ ≈ *H*. Therefore, t ≈ *W*/*H* and r ≈ *h*/*H*, which are referred to as the normalized ridge width and normalized outer ridge height.

On the premise of satisfying the calculation results of Equations (1) and (2), based on the reference data provided in reference [25], this group of data were selected according to the principle that the overall size of the pressure sensor is as small as possible. The data of the SOI ridge optical waveguide are as follows: *H* is 2 μm, *h* is 1.5 μm, and *W* is 5 μm, as shown in Table 1.

The higher the effective mode of the refractive index, the closer it is to the lower order mode and the more stable the transmission. Before optimization, the FEM method is used to find the appropriate effective mode refractive index.

**Figure 3:**
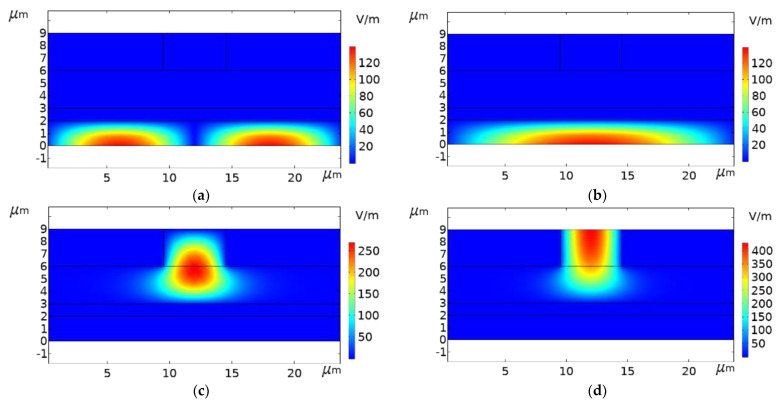
Surface: Electric field mode (**a**) effective mode refractive index N_1_ is 3.439; (**b**) N_2_ is 3.4395; (**c**) N_3_ is 3.44; (**d**) N_4_ is 3.4414.

Figure 3 expresses the electric field modes under different effective mode refractive indexes based on the core refractive index search. It can be seen that the electric field mode transmission is the most concentrated when the effective mode refractive index is 3.4414, so the final effective mode refractive index is 3.4414.

### 3.2. Optimization of a Ridge Optical Waveguide

After selecting the most appropriate effective mode refractive index, the directional coupler part in the ridge waveguide is optimized.

**Figure 4:**
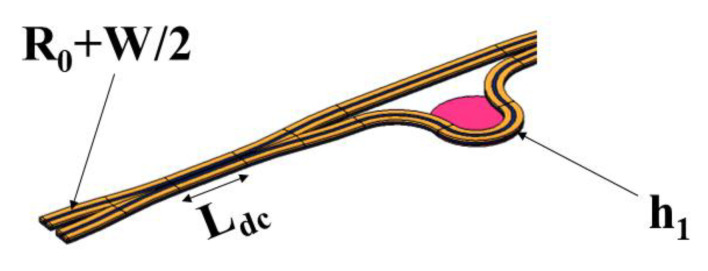
Main parameters of a ridge waveguide.

Figure 4 shows the main parameters of the directional coupler. R_0_ is the bending radius in the rib waveguide, l_dc_ is the waveguide length of the directional coupler in the rib waveguide, and *W* is the waveguide width.

**Figure 5:**
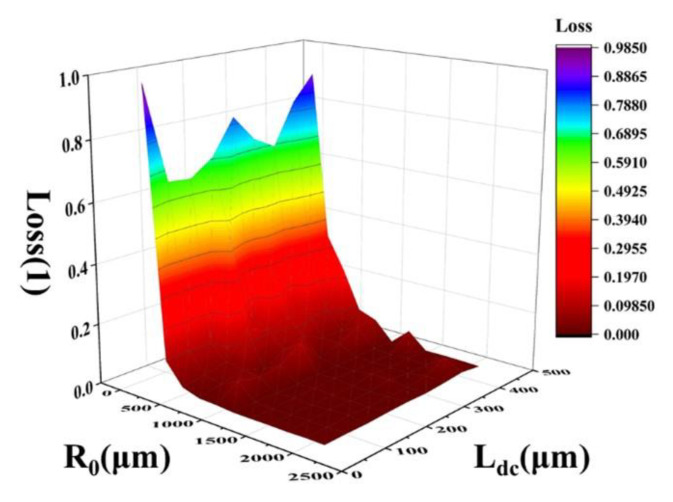
Influence of different bending radii and directional coupler waveguide lengths on transmission loss.

Referring to practical application scenarios, combined with the complexity of the structure, the cost and the difficulty of the processing technology, the influence of the bending radius in the range of 100–2000 μm and the waveguide length of the directional coupler in the range of 50–450 μm on the transmission loss is discussed. It can be seen from Figure 5 that, the larger the bending radius, the lower the output loss of the directional coupler. In the final selection of device parameters, the device size should be as small as possible, so the R_0_ is 500 μm.

**Figure 6:**
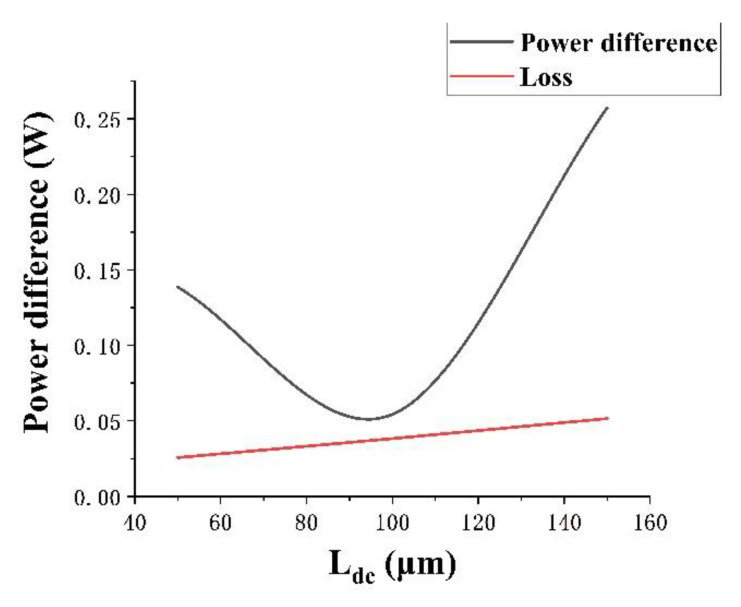
Effect of different waveguide lengths of the directional coupler on devices with a 500-μm bending radius.

In Figure 6, the black line represents power difference and the red line represents directional coupler device loss. When the waveguide length of the directional coupler is 100 μm, the overall loss is small, and the power difference is closest to 0, which meets the demand for the power sharing of the directional coupler.

According to the FEM simulation results, when the waveguide length of the directional coupler is 100 μm and the bending radius is 500 μm, the overall transmission loss of the directional coupler is 3.83%. When the input optical power is 1 W, the output power difference between the two output ports is only 0.05 W.

## 4. Design of an Elastic Film for a Pressure Sensor

The performance of a pressure sensor is affected by the material, size, and position of the elastic diaphragm. In terms of material selection, aluminum is a commonly used sputtering material in semiconductors, and it has good electrical conductivity and thermal conductivity. Processing technology can sputter and deposit the aluminum film under the sensing arm. Aluminum has high reflectivity, good surface performance, and low density for photothermal waves, which is suitable for mirror materials; aluminum is basically non-toxic and is suitable for biomedical applications. Therefore, aluminum was selected as the material of the elastic film. In addition, when the waveguide sensor is placed on the edge of the elastic diaphragm, it can produce the maximum phase change [26], so the diaphragm was placed at the overlap with the edge of the bending waveguide.

Since the size of the elastic diaphragm is related to the waveguide radius R’ of the middle part of the sensing arm, the waveguide radius is designed first.

**Figure 7:**
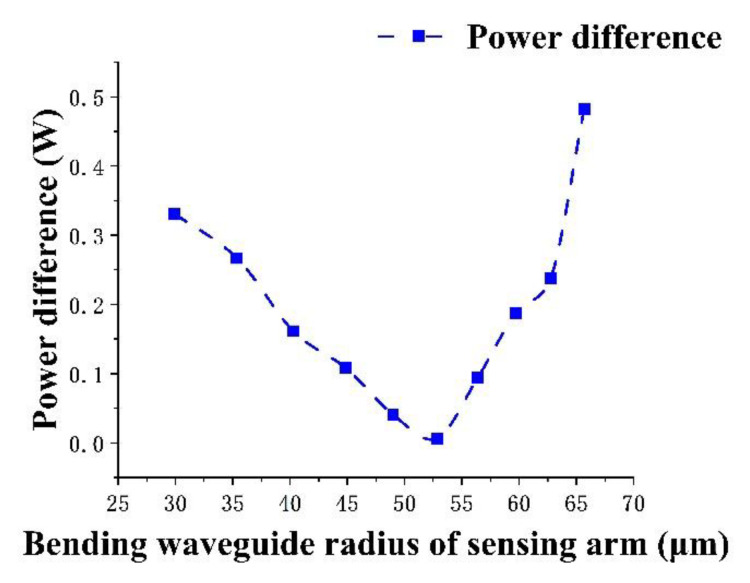
Effect of different R’ on the output power difference.

As shown in Figure 7, under the condition of no external light pressure, when R’ is 52 μm, the output power difference is the smallest. In order to observe the change between the two arms more clearly after external light pressure is applied, R’ is selected as 52 μm.

The optical input power is 200 GW/m^2^. According to the optical pressure Equation (4) [27], the maximum radiation pressure is 1.27 kPa, and the designed range of the pressure sensor is 0–1 kPa.

(4) $$P=\frac{I\left(1+R\right)}{c}$$

The formula *I* is 200 GW/m^2^ of light energy per unit time vertically incident to the unit area, *R* is 0.906 [28] of energy reflectivity of aluminum surface, *c* is 3 × 10^8^ m/s of light speed in a vacuum.

The maximum value of stress difference occurs at the middle of the edge of the diaphragm,

(5) $$\max\left(\left|\sigma_{x}-\sigma_{y}\right|\right)=\frac{1.23p_{1}\pi {R^{\prime }}^{2}\left(1-v^{2}\right)}{h_{1}{}^{2}}\;\leq \frac{1}{5}\sigma_{m}$$

where *p*_1_ is 1 kPa of the design range, *v* is 0.33 of the Poisson ratio, and *h*_1_ represents the thickness of the elastic film, as shown in Table 2.

From Equation (5), *h*_1_ ≥ 2.04 μm. Next, based on the research idea of reference [29], by studying the displacement variation trend of different film thickness under the same external light pressure and the phase difference of different film thicknesses at the same position under 0–1000 Pa external light pressure, the most suitable film thickness was selected.

**Figure 8:**
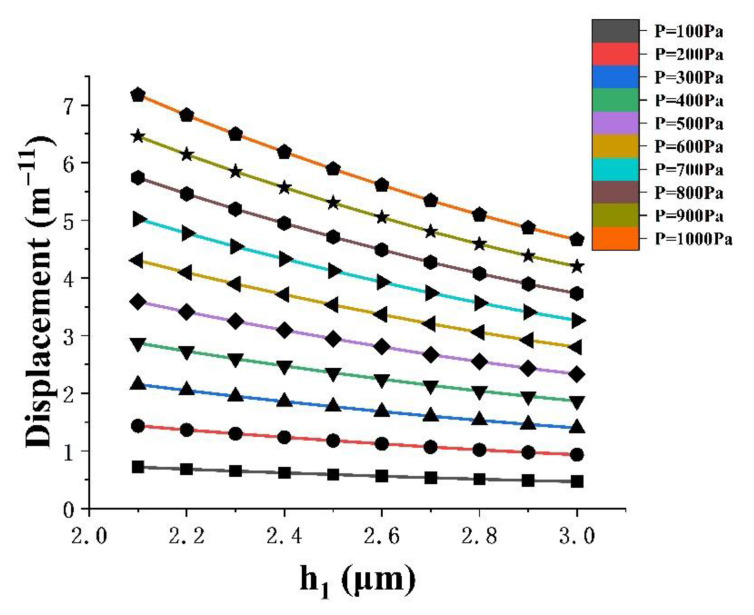
The displacement variation trend of different film thicknesses under the same external optical pressure.

**Figure 9:**
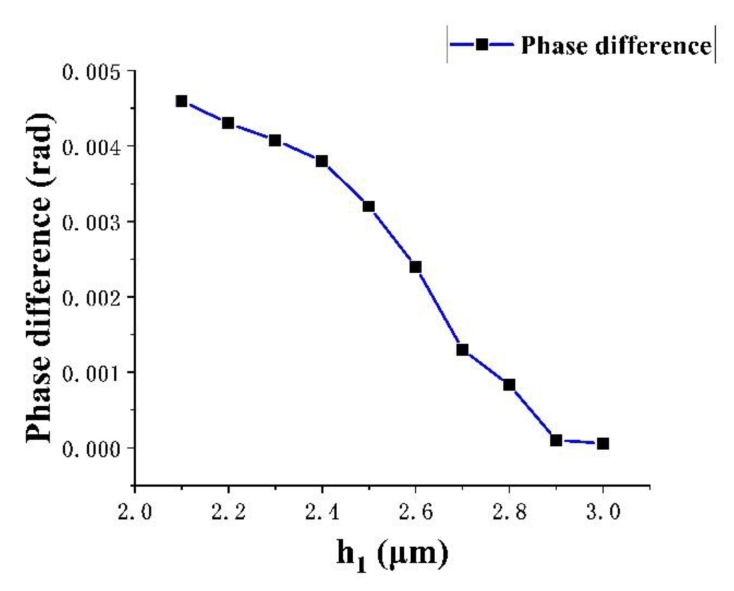
The phase difference of different film thicknesses at the same position under a 0–1000 Pa external optical pressure.

According to Figure 8, for the same position, under the same applied light pressure, the smaller the film thickness, the larger the deformation displacement; in the range of 0–1000 Pa, the smaller the film thickness, the greater the deformation. It can be seen from Figure 9, that for the same position, within the range of a full-scale input, the smaller the film thickness, the larger the phase change amplitude. Therefore, in order to make the pressure sensor have a better linear output performance and higher sensitivity, the film thickness was selected as 2.1 µm.

**Figure 10:**
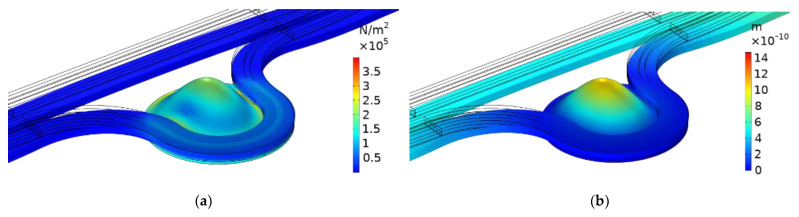
(**a**) Stress distribution at the center of the device, when the diaphragm thickness is 2.1 μm; (**b**) Displacement distribution of the device center at 2.1 μm of diaphragm thickness.

Figure 10 is the stress and displacement nephogram when the film thickness is 2.1 μm and when the applied load is full range. It can be seen in Figure 8 that the film thickness of *h*_1_ = 2.1 μm met the strength requirements.

## 5. Performance Analysis of the Pressure Sensor

The main factor influencing the variation of the output intensity of the pressure transducer, considering a loss-free condition, is the phase difference between the two arms.

(6) $$I_{out}=I_{in}cos{\left(\frac{\Delta \varphi }{2}\right)}^{2}$$

The phase difference between the two arms is calculated according to the following equation [20]:

(7) $$\Delta \varphi =\frac{2\pi }{\lambda_{0}}L_{0}\Delta n_{eff}+\frac{2\pi }{\lambda_{0}}n_{eff}\Delta L+const$$

where $\lambda_{0}$ is 1550 nm, the sensing arm length *L*_0_ is 3.93 × 10^−4^ m, and ∆L is the length change of the sensing arm under different pressures. The specific value is approximately selected as the displacement change of the contact position between the sensing arm and the elastic diaphragm in the simulation. The numerical change is shown in Figure 9. Δ*n_eff_* is the difference between the effective mode refractive index and the previous N_4_ under different pressures. As the external light pressure is small, the value of ∆*n_eff_* at four positions remains unchanged after the decimal point, and its value is 3.4414 − 3.4398 = 0.0016. const is 1.43 × 10^−4^ m, which is the length difference between the two arms. The parameters used in phase difference calculation are summarized in Table 3.

**Figure 11:**
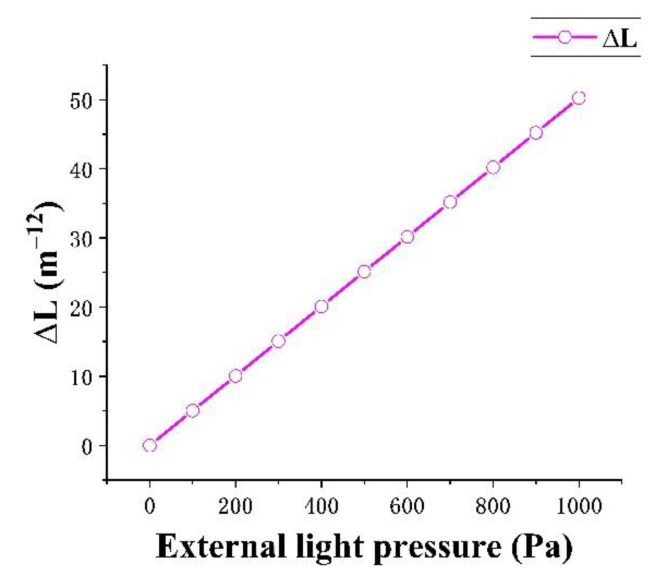
Relationship between ∆L and external light pressure.

Combined with the pressure sensor structure diagram and Equations (6) and (7), the main factor affecting the phase difference change between the two arms is the change in the refractive index of the material sensing arm caused by the applied load, which leads to the change of the phase difference between the two arms. Therefore, the FEM solution was used to simulate the change of phase difference between two arms with pressure, and the results were compared with the theoretical formula, as shown in Figure 11.

**Figure 12:**
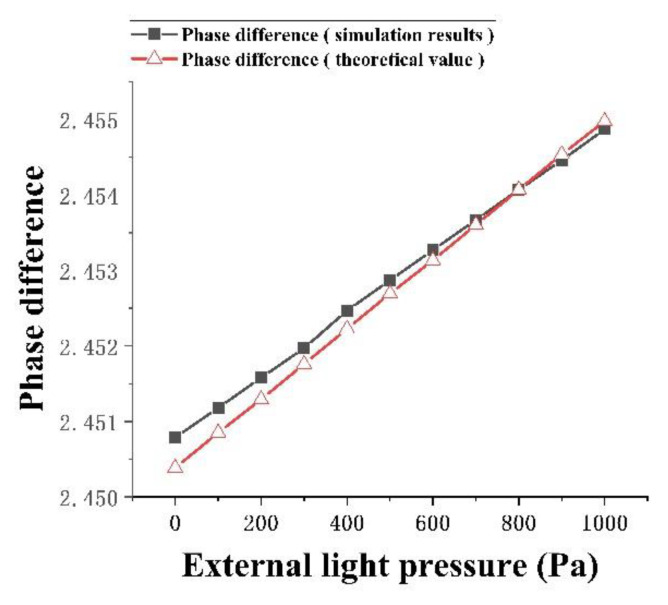
Relationship between phase difference of two arms and external light pressure.

It can be seen from Figure 12 that the simulation results were close to the theoretical results. As the applied load increases gradually, the phase difference between the sensing arm and the reference arm of the pressure sensor also increased gradually, which affected the output strength of the pressure sensor. In the simulation, the position selected to study the change of phase difference was the place where the stress deformation of the sensing arm is the largest. Compared with the theoretical results, the research scope of the simulation results did not cover the whole sensing arm, so the conclusions of the two are slightly different. However, according to Figure 10, the difference between the two was very small and did not affect the subsequent simulation research.

### 5.1. Pressure Sensor Sensitivity and Linearity Analysis

Sensitivity is the output produced per unit of input under static operating conditions and is expressed by the equation

(8) $$\underset{\Delta x\to 0}{lim}\frac{\Delta y}{\Delta x}=\frac{dy}{dx}$$

The input optical power in this paper is 1 W, based on FEM simulation results according to the output power value under different pressures (*P_in_*); The relationship curve of the port output power value (*P_out_*) with the change of pressure is determined, and then the curve is fitted by a straight line. The slope of the curve is the sensor sensitivity *S*

(9) $$S=\frac{\Delta p_{\mathrm{out}}}{\Delta P_{\mathrm{in}}}$$

**Figure 13:**
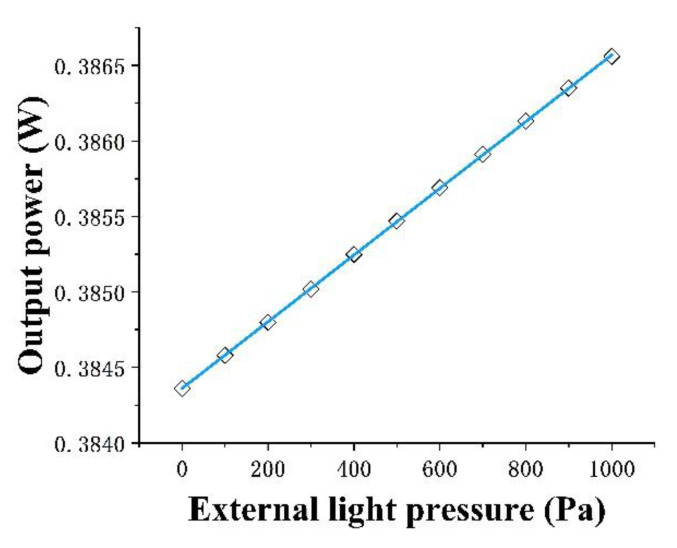
Relationship between port output power and pressure (black dots are simulation data, red lines are linear fitting).

Figure 13 shows the scatter plot of the output power at different pressures, obtained by simulating using FEM simulation and the resulting plot obtained by linear fitting using origin software. According to Figure 10, it can be concluded that the sensitivity of the pressure sensor was 2.2 × 10^−3^ W/kPa at an input optical power of 1 W.

Since sensor linearity is the maximum deviation of the sensing calibration curve from the fitted straight line as a percentage of the full-scale output, the linearity of the pressure sensor could be calculated from Figure 10 as 5.9 × 10^−3^.

### 5.2. Technological Process of Pressure Sensor

The fabrication process of the pressure sensor mainly entailed of lithography and deposition. The chemical vapor deposition (CVD) process was used for silicon dioxide thin films. First, the ridge structure was etched using lithography, and then the silicon dioxide thin films were deposited using the CVD process. Plasma enhanced chemical vapor deposition (PECVD) technology was used in preparation of aluminum film. The technology used for the aluminum film was the evaporation process (PECVD). Since the thickness of the silicon substrate of SOI is usually more than 300 μm, if the holes with the same radius as the elastic film and the thickness of the silicon substrate are selected as the prerequisite for evaporation, it can be difficult to guarantee the perpendicularity and cylindricity of the elastic film during evaporation, and it is difficult to observe whether the aluminum film has fallen off. In order to solve this problem, photolithography was performed twice before the evaporation process, the first photolithography retained a thickness of 50 um for the Si substrate, and the hole size was greater than the elastic film area. On the basis of the first lithography, the second lithography etched holes with the same radius and depth of 50 um as the elastic film, which could better ensure the verticality and cylindricity of the elastic film in the subsequent evaporation process, and also facilitate the subsequent observation of the aluminum film. The process roadmap is shown below.

**Figure 14:**
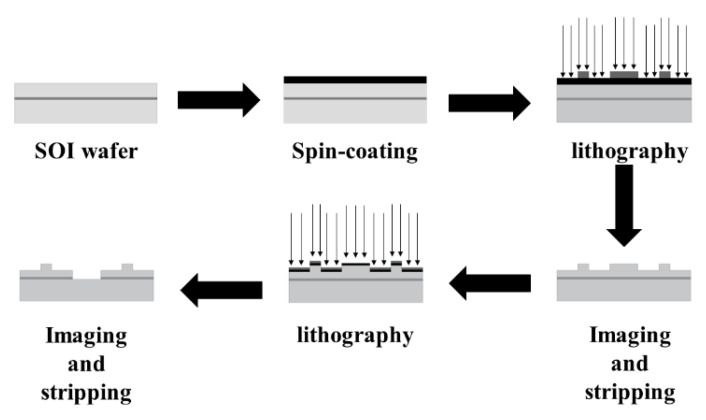
MEMS technological process.

**Figure 15:**
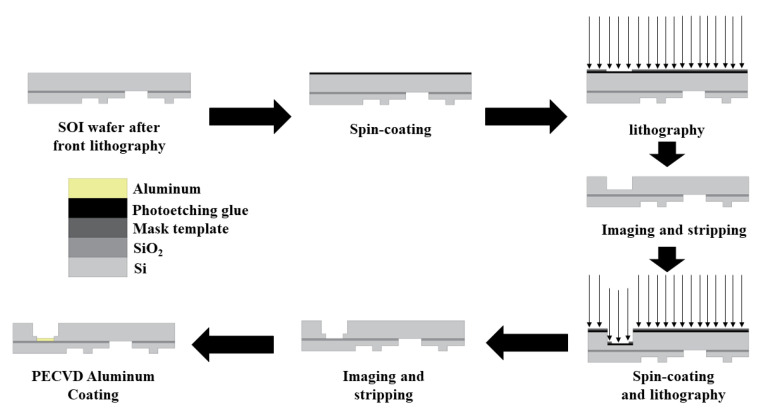
MEMS technological process (back cavity).

The designed pressure sensor can be fabricated according to the process route shown in Figure 14 and Figure 15.

## 6. Conclusions

A ridge waveguide pressure sensor based on MZI was designed and combined with SOI material. When the input light power was 1 W, the sensitivity was 2.2 × 10^−3^ W/kPa and the linearity was 5.9 × 10^−3^. Since the working mechanism of the optical sensor is to change the phase of the transmitted laser through the change of the refractive index in the waveguide, the optical pressure sensor is not affected by electromagnetic field interference. In comparison to traditional mechanical pressure sensors, it has the advantage of being small and easy to integrate, and at the same time can measure small pressures with high sensitivity and linearity, and could be used in the field of light pressure measurement and other fields that require measuring small pressures, such as the biomedicine field.

## Figures and Tables

**Table 1:** Geometrical parameters of the ridge structure.

Item	Value	Unit
Coefficient constant (*c*_1_)	0.3	None
Ridge width (*W*)	5	μm
Ridge height (*H*)	2	μm
Outer ridge height (*h*)	1.5	μm
Refractive index of cladding (*n*_1_)	1.445	None
Refractive index of core (*n*_2_)	3.445	None
Refractive index of insulating layer (*n*_3_)	1.445	None

**Table 2:** Parameters for Calculation of Elastic Film Thickness.

Item	Value	Unit
Optical input power (*I*)	200	GW/m^2^
Design range (*p*_1_)	1000	Pa
Poisson ratio (*v*)	0.33	None

**Table 3:** Parameters for phase difference calculation.

Item	Value	Unit
Length of sensing arm (*L*_0_)	3.93 × 10^−4^	m
Length difference between the two arms (*const*)	1.43 × 10^−4^	m
Effective mode refractive index in simulation (*n_eff_*)	3.4398	None
Wave length (*λ*_0_)	1550	nm

## Data Availability

Not applicable.
